# Challenges in using pupil dilation responses to sounds as a reliable alternative to standard audiometric tests

**DOI:** 10.1016/j.heliyon.2025.e42666

**Published:** 2025-02-14

**Authors:** Maria Paola Tramonti Fantozzi, Antonino Crivello, Davide La Rosa, Mario Milazzo, Serena Danti, Vincenzo De Cicco, Paolo Orsini, Diego Manzoni, Francesco Lazzerini, Rachele Canelli, Giacomo Fiacchini, Luca Bruschini

**Affiliations:** a Department of Translational Research and of New Surgical and Medical Technologies, University of Pisa, Pisa, Italy; b Institute of Information Science and Technologies, National Research Council of Italy, Via Giuseppe Moruzzi, 1, Pisa, 56127, Italy; c Department of Civil and Industrial Engineering, University of Pisa, Pisa, Italy; d The BioRobotics Institute, Scuola Superiore Sant'Anna, Pontedera, Pisa, Italy; e Department of Surgical, Medical, Molecular Pathology and Critical Cares, University of Pisa, Pisa, Italy

**Keywords:** Audiometry, Auditory detection, Orienting reflex, Pupil dilatation, Pupillometry

## Abstract

Assessing hearing in neonates and uncooperative patients can be challenging. Pupil dilation response (PDR) as an objective physiological measure may offer a solution. To test its feasibility, PDRs were averaged in response to a sequence of 60–100 audible tones (same frequency and amplitude). This was performed in subjects with normal hearing and communication abilities, who were exposed to two different lighting levels. We evaluated whether averaged post-stimulus intervals of PDRs were significantly different from randomly generated averages of pupil traces recorded in the absence of sound stimulation from the same subject. This analysis was repeated for the first, second, and third block of PDRs to account for possible adaptation phenomena. Although all the participants clearly perceived the tones, significant PDRs in response to sound were only detected in a fraction of subjects, primarily in the low luminance condition. Consequently, only in the low luminance group, the grand average of individual PDRs was significantly larger than that obtained for traces recorded in the absence of sound input. In this most favorable condition, when the three blocks of PDRs were averaged separately, significant PDRs were observed in 40 % of the subjects in at least one of the blocks. Therefore, the PDR to sound input is not a reliable indicator of hearing perception when standard audiometric stimuli of the same amplitude and frequency are used. Possible modifications to sound input and stimulation protocols for obtaining reliable PDRs in diagnosing and treating hearing impairments are discussed.

## Highlights

• Our study explores Pupil Dilation Response (PDR) as an objective hearing measure in neonates and uncooperative patients.
• We exposed healthy subjects to tones and compared PDRs to baseline pupil traces to determine their significance.
• Significant PDRs to sound were mainly observed in subjects under low luminance conditions.
• PDR was not consistently reliable for hearing perception using standard audiometric stimuli of the same amplitude/frequency.

## 1 Introduction

Human hearing is typically assessed by evaluating motor responses or obtaining verbal feedback from cooperative patients, which helps to determine their ability to detect pure tones of varying amplitudes and frequencies. However, this approach is not always practical, especially in cases involving neonates or pre-lingual infants. In such instances, early hearing assessments become essential, as the presence of auditory neuropathy may necessitate early interventions, like hearing aids or cochlear implants, to optimize their communication development [[1], [2], [3]]. Another challenging context where reliance on verbal or motor responses to sound is problematic is dementia. Although mild cognitive impairment does not necessarily hinder accurate audiologic assessment [4], standard audiometric tests cannot be completed by 41%–44 % of dementia patients [5]. This challenge has spurred the development of complementary non-behavioral hearing tests [5]. In such situations, electrophysiological auditory tests, like the auditory brainstem response (ABR) or the auditory steady-state response (ASSR) [6], can be used to assess hearing thresholds. However, for children and uncooperative patients, these tests often require sedation or must be conducted during spontaneous sleep [6].

Based on the results of literature studies, the big clinical challenge that scientists have to address concerns the development of alternative approaches to standard audiometric tests, able to provide a reliable assessment of the hearing capabilities of subjects, independently of their specific ability to relate to clinicians.

A potential solution to this task may involve the recording of objective physiological variables related to sound perception. Specifically, a sudden sound input or a change in a regular sequence of identical acoustic stimuli triggers a shift of the attention to the unexpected event which is indicated as “orienting response [7,8]. The orienting response includes a pupil dilation (PDR, pupil dilatation response), which amplitude is influenced by various factors [9], including the cognitive resources allocated to the perceptual task [10]. A gaze shift takes also place when the stimulus is lateralized [11]. The PDR has been considered a measure of the effort associated with a listening task [12], being more pronounced when auditory perception is more demanding [13]. Pupil size traces recorded in response to sound input have demonstrated that the PDR is elicited by different types of sound, including pure tones [9] and is associated with sound detection in both animal models [14,15] and humans [10,16,17]. A PDR has been also observed in both monkeys and humans during an acoustic oddball in response to rare stimuli [18].

Although it could be argued about the difficulty to obtain orienting of attention and gaze in the categories of patients mentioned above, a PDR has been documented in pre lingual children submitted to sound presentation while attending to visual stimuli [19,20]. Within this population, during an acoustic oddball paradigm where the rare stimuli consist in unexpected sounds, a PDR can be obtained in response to the latter ones [21,22], particularly when they have an emotional content [22]. A PDR can be also observed in people affected by dementia [[23], [24], [25]].

Therefore, PDR is a promising method for investigating hearing in individuals with limited communicative abilities. However, its reliability in the assessment of hearing ability depends on the robustness of its association with hearing perception at single subject level. Moreover, due to the spontaneous fluctuation of pupil size and to the small amplitude of the PDR the definition of this response must include a statistical evaluation of its significance [17]. The PDR has been extensively studied at the population level [10,16,17], but to the best of our knowledge, only two papers [17,18] have shown single subject traces and only one has established their statistical significance [17]. Moreover, none of these studies reported whether the association of PDR with hearing perception took place in all the subjects or could be detected only in a percentage of them. This finding needs to be clarified using the standard pure tone stimuli commonly employed in audiometric tests and has yet to be assessed in a population of normal subjects. In any event, developing a test of hearing perception not including a subjective report from the patient may require further steps. It is in fact necessary to utilize stimuli appropriate for retrieving a (almost) one-to-one correspondence with perception, and tailored to the characteristics of the target population. Moreover, a parallel decrease of PDR and sound amplitude until the perceptual threshold is reached must be documented.

In this study we conducted preliminary investigations in normal subjects to assess evidence of reliable PDRs when individual subjects correctly perceived a standard audiometric supra-threshold stimulus.

## 2 Materials and methods

### 2.1 Subjects

The Ethical Committee of the Pisa University (Italy) approved the study with the protocol labeled 45/2023 on December 5, 2023. The study was carried out on 16 volunteer healthy subjects (i.e., 9 females; 7 males), endowed with normal hearing ability, and mean age 30 ± 4 years. The recruitment period for this study was between December 6, 2023, and December 12, 2023. The study was conducted in accordance with the Declaration of Helsinki; as such, the participants were properly informed and signed a written informed consent in presence of at list one of the co-authors of this work. All documents were properly archived by the Pisa University (Italy).

Each participant sat comfortably on a chair within a room with controlled lighting conditions similar to those normally observed in clinical rooms. The room illumination was maintained within two distinct, non-overlapping luminance ranges: from 8 to 12 lux (referred to as Low Luminance, LL) and from 95 to 115 lux (referred to as High Luminance, HL). Luminance levels were measured using a light meter (MT-912, Shenzhen Flus Technology Co. Ltd, Pinghu Town, Longgang District, Shenzhen, China). In total, 6 subjects were examined under LL conditions, 6 under HL conditions, and 4 experienced both luminance conditions.

Participants wore eye tracking glasses (Pupil Labs GmbH, Berlin, Germany), enabling simultaneous recording of pupil size before, during, and after sound stimulation.

Participants were instructed to sit back and relax, looking straight ahead, as is customary during standard audiometric tests. However, they were not required to focus on a specific target in front of them. Pupil size data for each subject were initially recorded continuously in the absence of sound stimulation for 8 min (i.e., referred to as the Baseline condition). Subsequently, the acoustic stimulation was administered every 4 s for 8 min (i.e., referred to as the Audio condition). Overall, each subject was submitted to 120 stimuli, which were delivered through a headset connected to an AC 40 audiometer (Interacoustics, Middelfart, Denmark), commonly employed in clinical settings and supervised by a specialized technician. The acoustic stimulus consisted of a 2000-Hz pure tone with an amplitude of 70 dB HTL and 500 ms duration, which was well-perceived by the subjects. Since the stimulus intensity was the same for both headphones, the stimulus was localized at the center of the head. The stimulus was clearly audible for all the subjects investigated.

### 2.2 Eye tracking glasses and pupil size recording

To continuously measure pupil size, we employed the Pupil Core eye tracking glasses developed by Pupil Labs GmbH in Berlin, Germany. These glasses are connected to a laptop via a USB connection and are equipped with two Infrared (IR) cameras for real-time eye imaging. Additionally, they featured an integrated visible light camera mounted on the top bar of the glasses to capture the user's point of view. In the post-processing phase, we used the Pupil Player software tool that comes with the glasses to process the recorded videos.

This software identified the shape of the pupils, thus calculating their diameters in pixels with the help of a 3D model of the eyeball correcting the distortion introduced by the camera perspective, and providing a confidence index within the range of 0–1. This index represented the algorithm reliability score for pupil shape detection. Following the manufacturer's recommendations and based on empirical testing, we adopted a confidence threshold of 0.8 to classify a measurement as reliable. The tool also detects blinking events, defined as time intervals during which the pupils are partially or fully obstructed by the eyelids.

In general, each session generated two datasets - one for the left pupil and one for the right pupil. Each dataset included the time series of the parameters presented in Table 1, sampled for each frame of the video recording, with an approximate frame rate of 62 FPS, for an overall recording period of 8 min.

**Table 1:** Data extracted from each frame of the pupil video recordings.

Name	Range of values	Description
**Confidence**	0.0 (worst) - 1.0 (best)	Parameter representing the reliability score given by the algorithm to the pupil shape detection
**Diameter**	>0	Diameter of the pupil, expressed in pixel, estimated by the algorithm
**Blink**	0 (no)/1 (yes)	Whether the eyelid partially of fully covers the pupil area
**Artifact**	0 (no)/1 (yes)	Whether the tracking of the pupil shape is missed and it is not caused by a blinking event
**Audio**	0 (no)/1 (yes)	Whether the acoustic stimulus is present

**Fig. 1:**
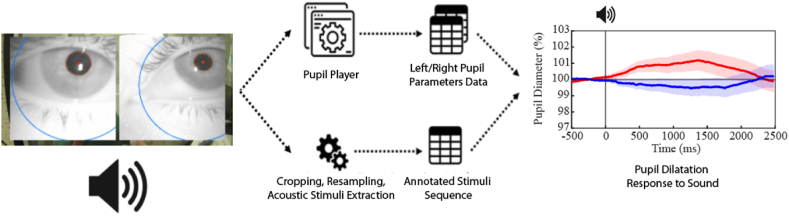
**Pipeline showing the steps required to acquire the video recordings, extract and subsequently merge the data of both the pupil parameters and the acoustic stimuli.** The red and blue traces show the average values of Audio and Baseline (no stimulus) frames, respectively.(For interpretation of the references to color in this figure legend, the reader is referred to the Web version of this article.)

We utilized the additional camera on the glasses to record the visual feedback provided by the audiometer, indicated by an LED blinking on the instrument when the operator triggered the impulse. A visual barrier was used to prevent subjects from seeing the LED activation, thereby avoiding potential conditioning of their responses to the auditory stimuli. During the post-processing phase, the recordings from the environmental camera were cropped, resampled at 120 FPS, and analyzed to extract the time instants at which the acoustic stimuli were administered to the subjects. These time instants were aligned with the time series of pupil size obtained from the two IR eye cameras. The data acquisition and processing pipeline is outlined in Fig. 1. This rather complex procedure was necessary to synchronize the acoustic stimulus with the pupil recording (it was not possible to use the sound signal as a trigger for pupil size recording).

The generated datasets, which contained the time-synchronized traces of both the pupils' parameters and the acoustic stimuli, were then forwarded to the subsequent stage for further processing.

### 2.3 Processing of pupil size traces

Since achieving a highly reliable pixel-to-mm conversion factor would have required an *ad-hoc* calibration for each individual participant, which is challenging to implement in the clinical environment where audiometric experiments were conducted, pupil size data were instead acquired in pixels to maintain consistency and reliability across recordings. As the generated traces are a result of merging multiple video sources, the final sampling frequency was not constant. Therefore, all Baseline and Audio pupil data were resampled at 50 Hz using custom software implemented within a LabVIEW environment provided by National Instruments Corp., (Austin, Texas, USA). Data points corresponding to moments identified as blinks or artifacts with a confidence score below 0.8 were replaced by the software with a linear interpolation based on the nearest and reliable data points. The effective removal of the artifacts was verified by visual inspection of the individual traces.

Both the Audio and Baseline data were detrended and segmented into frames of 3 s (150 points). Frames derived from the Audio trace included a pre-stimulus period of 0.5 s, while those from the Baseline trace were chosen to correspond to the same time instants where the auditory stimuli would have occurred during the Audio condition. We remark that in the Baseline condition neither audio nor visual (through the LED) stimuli were administered to the subjects Frames containing more than 15 points corrected by linear interpolation were discarded from the analysis. Data within each frame were expressed both as a percentage and as a difference with respect to the average value of the initial 25 data points (equivalent to 0.5 s), which corresponded to the pre-stimulus period in the Audio traces.

Subsequently, the left and right pupil frames of individual subjects were averaged. Baseline and Audio traces were treated separately. This allowed us to obtain an average Audio and an average Baseline trace of pupil diameter, which could be statistically compared to identify the presence of PDRs in the single subjects. Raw data have also been published in Ref. [26].

### 2.4 Statistical analysis

The first goal of the study was to assess the reliability of the test in establishing the occurrence of sound perception by the subject. The sound amplitude was well above the hearing threshold in all the subjects, so that a clear PDR should have been detected in each participant. PDR occurrence was evaluated by comparing Audio and Baseline pupil frames of each participant dataset [17], obtained by pooling together left and right pupil data. In the Audio frames, where sound stimulation begins at 0.5 s, a PDR is expected to occur in the 0.5–2.5 s interval, while in the Baseline traces this period contains only random fluctuation of pupil size. The criterion for defining the occurrence of a significant PDR was therefore the presence of a significant difference between Audio and Baseline frames in the interval 0.5–2.5 s. To perform this comparison, in each single frame we evaluated the area enclosed between the pupil trace and a line corresponding to the average value (100 %) of the 0–0.5 s interval. In the Audio frames this interval represents the pre-stimulus period. When pupil sizes were below 100 %, we considered the area with a negative sign. The area was evaluated in various post-stimulus intervals: 0.5–1.0 s, 1.0–1.5 s, 1.5–2.0 s, and over the entire 0.5–2.0 s period.

We identified significant PDRs when the area of Audio frames within one or more intervals was significantly larger than that in the Baseline frames, as determined by an unpaired, two-tailed *t*-test.

A further analysis was conducted in few subjects where more than one data set could be recorded and the effect of increasing the number of averaged traces was observed.

Finally, the effect of luminance level on PDR was evaluated. Since only a few subjects could be tested for both LL and HL conditions, due to limited compliance of the participants, a paired data comparison model could not be applied. For each luminance group, we performed grand averages of Audio and Baseline data by pooling together all the frames obtained in all the subjects. These Audio and Baseline grand averages were compared based on the post-stimulus areas of individual traces, following the same criteria as used for individual subjects. For this purpose, an unpaired, two tailed *t*-test was utilized. In this analysis the degrees of freedom corresponded to “nA + nB −2”, where nA and nB represent the total number of averaged Audio and Baseline traces, respectively. The significance level was set at *p* < 0.05. Comparisons between linear regression coefficients were performed by the test of equity for unpaired data [27], while comparisons of the corresponding according to Cohen et al. [28].

## 3 Results

Within the total sample consisting of 16 subjects, 6 underwent testing under high luminance, 6 under low luminance, and 4 experienced both luminance conditions. As a first step we evaluated the presence of significant PDRs in individual subjects, by comparing average Audio and Baseline frames, as reported in the Methods.

Averages were calculated using 65–117 Audio traces and 82–116 Baseline traces. The variability in the number of frames averaged was due to the presence of too many blinks and eye movements artifacts within a fraction of the frames (see Methods). The analysis was performed on data normalized both as a percentage and difference with respect to the average values of the pre-stimulus interval. The results obtained with the two datasets were strictly corresponding, so that only those relative to percentages will be described.

**Fig. 2:**
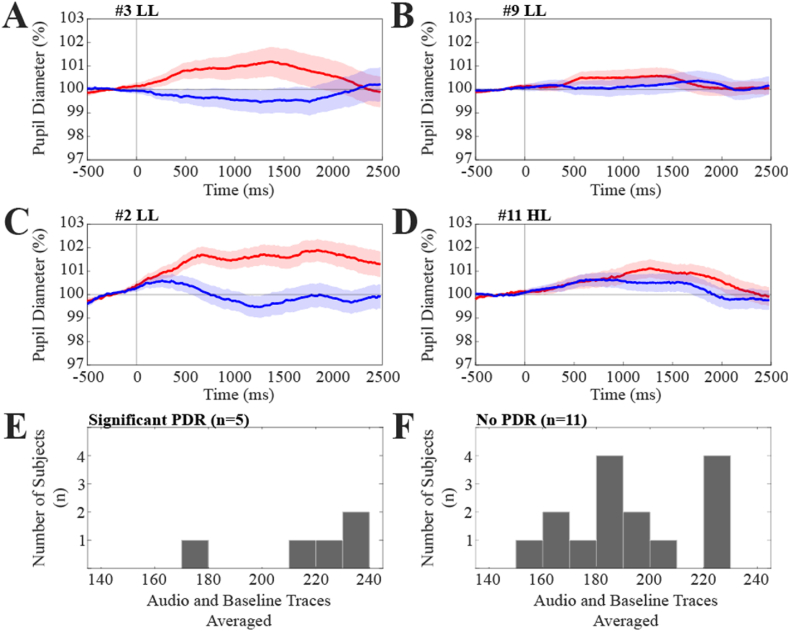
**Examples of pupil dilatation responses in individual subjects.** Individual average pupil dilatation responses to acoustic stimulation (Audio traces, red) are shown in two subjects (A and C). The plotted values correspond to the pupil size expressed in % of the pre-trigger value (average value of the −500/0 interval). These responses were compared to the corresponding Baseline (blue) traces, which represent pupil size recorded in the absence of acoustic stimulation. In both A and C, significance was documented in the intervals 0.5–1.0 s, 1.0–1.5 s, 1.5–2.0 s, as well within the whole 0.5–2.0 s period. B and D show two subjects which individual average pupil responses to sound were not significantly different from the corresponding Baseline traces. The colored regions around average traces correspond to Standard Error (SE) values. The last row shows the distribution of the number of cumulative Audio and Baseline traces utilized to assess pupil responsiveness to sound in the subjects showing (E) and not showing (F) significant PDRs, respectively.(For interpretation of the references to color in this figure legend, the reader is referred to the Web version of this article.)

We observed a significant difference between Audio and Baseline pupil size traces (indicative of a significant PDR within the entire 0.5–2.0 s interval or in some of its 0.5 s partitions in 3 out of 10 subjects (i.e., 30 %) tested under low luminance conditions and 2 out of 10 subjects (i.e., 20 %) tested under high luminance conditions. Significance was assessed, as indicated in the Methods, by comparing the areas included between the pupil traces and the average pre-trigger value. Examples of subjects with (A, C) and without (B, D) pupil responses to sound are presented in Fig. 2, along with the distribution of the number of pupil traces averaged in the two groups of subjects showing presence (E) and absence (F) of PDR. Fig. 2 also shows that the number of averaged Audio and Baseline frames utilized in the test was comparable in subjects that displayed (E) or did not show (F) significant PDR.

Table 2 summarizes data for all subjects displaying post-stimulus intervals with significant differences between Audio and Baseline traces. It's worth noting that the increases in pupil size associated to PDRs were of very small amplitude, ranging from 0.9 % to 2.7 % of the pre-stimulus average value.

**Table 2:** Characteristics of pupil size response to sound observed in individual subjects.

Sub (#)	Lum	Int (s)	Audio Traces (n)	Pupil Size Audio Traces (%)	Baseline Traces (n)	Pupil Size Baseline Traces (%)	*p*-value
**1**	LL	0.5–2.0	92	101 ± 6	79	99 ± 4	0.007
**2**	LL	0.5–2.0	117	102 ± 4	118	100 ± 4	0.001
**3**	LL	0.5–1.0	113	101 ± 5	101	100 ± 4	0.029
**4**	HL	0.5–1.0	114	103 ± 6	112	101 ± 5	0.007
**2**	HL	1.0–1.5	116	101 ± 4	116	100 ± 3	0.030

Note: Average ± SD pupil size values of Audio and Baseline frames evaluated for each subject displaying significant differences between the two data sets, as defined in the Methods. Data have been evaluated over the time interval where the difference was assessed (Int), its significance being reported in the last column on the right (p-value). LL: low luminance level. HL: high luminance level. The p-value refers to the comparison of the average Audio and Baseline pupil size evaluated in the corresponding intervals.

**Fig. 3:**
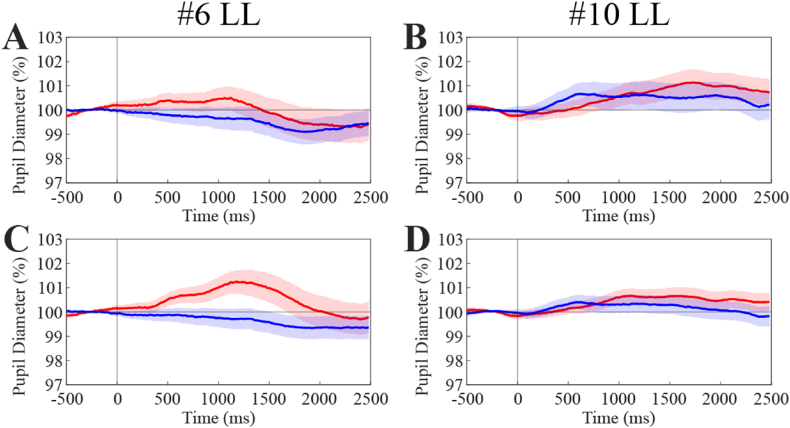
**Effect of increasing averaged traces for detection of pupil dilatation response to sound.** The upper row represents initial lack of pupil responsiveness to sound in two different subjects following averaging 164 (A: Audio, n = 65; Baseline, n = 99) and 191 (B: Audio, n = 97; Baseline, n = 94) cumulative traces. C. Same subject as in A following averaging of 265 (Audio, n = 112; Baseline, n = 153) cumulative traces. D. Same subject as in B following averaging of 346 (Audio, n = 174; Baseline, n = 172) cumulative traces. Red and blue colors represent Audio and Baseline traces. The colored regions around average traces correspond to SE values.(For interpretation of the references to color in this figure legend, the reader is referred to the Web version of this article.)

Repetition of the testing session could be performed only in two subjects of the low luminance group, who did not show significant Audio/Baseline differences following the averaging of left and right pupil traces. They underwent further recordings and averaging of left pupil responses to sound, as well as relative Baseline traces (Fig. 3). Only one of them displayed a significant Audio-Baseline difference when additional traces were averaged (Fig. 3A, C).

**Fig. 4:**
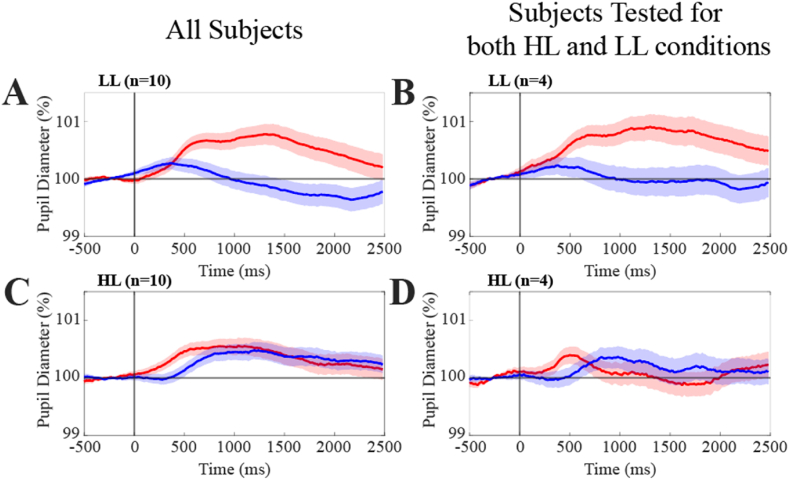
**Grand average of Audio and Baseline pupil traces under low and high luminance conditions.** Average pupil response to sound in low (A, B) and high (C, D) luminance conditions. Audio and Baseline traces correspond to red and blue lines, respectively. The colored regions around average traces correspond to SE values. In A and C, the grand average was performed over all the recorded pupil size traces of all the subjects analyzed, while in B and D it was restricted to the traces relative to the four subjects tested under both luminance conditions.(For interpretation of the references to color in this figure legend, the reader is referred to the Web version of this article.)

Following these individual subject analyses, a grand average, based on the start of tone presentation, was calculated for all individual subjects' right and left pupil Audio frames in both luminance groups (LL: n = 942; HL: n = 1037). Baseline frames (LL: n = 958; HL: n = 1036) were similarly processed. These results are presented in Fig. 4.

As shown in Fig. 4A, in the low luminance group a significant difference was observed between Audio and Baseline grand averages within all considered intervals (0.5 – 1.0 s, 1.0 – 1.5 s, 1.5 – 2.0 s, 0.5 – 2.0 s, p < 0.001). It has to be underlined, however, that the peak of pupil dilatation observed for the audio frames was only about 1 % of the pre-trigger value. No significant differences were observed under high luminance conditions between Audio and Baseline grand averages, regardless of the interval considered (Fig. 4C). Similar results were obtained when the analysis was limited to the four subjects tested under both luminance levels, despite the limited sample (Fig. 4B, D). It is worth of note that a great between-session difference in pre-stimulus pupil diameter, which largely overwhelmed the uncertainty of the pixel-to-mm conversion factor, was observed for the 4 subjects tested in both conditions (HL: 68.8 ± 17.2 px; LL: 100.6 ± 26.2 px, *p* = 0.033).

### 3.1 PDR and pre-stimulus pupil size values

**Fig. 5:**
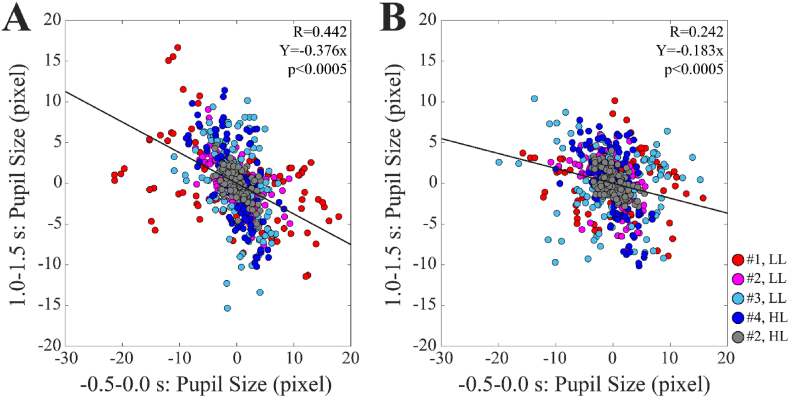
**Correlation of PDR with the corresponding pre-stimulus values of pupil diameter.** Panel A shows the correlation between the PDR and the corresponding pre-stimulus values obtained for all the frames relative to the four subjects shown in Table 2. Both variables were expressed as the difference with respect to the mean values of the individual subjects. Panel B shows that a similar, but significantly weaker correlation was found for the same subjects when the stimulus was absent and comparable amounts of equally spaced frames were randomly selected from the Baseline trace (see text).

The possible dependence of PDRs (recorded in the 1.0–1.5 s interval) upon the pre-stimulus pupil size values was investigated for the 5 responses shown in Table 2. Significant negative correlations were observed between these two variables for all the subjects. Such a correlation could be sometimes observed also when the stimulus was absent (3 out of 5 responses) and comparable amounts of equally spaced frames were randomly selected from the Baseline trace. In this case, however, the regression lines tended to show smaller slopes with respect to those obtained from Audio frames. This difference became evident when data from different subjects were pooled together. For this purpose, both variables were expressed as the difference with respect to the individual mean values. This analysis is displayed in Fig. 5, which shows the significantly larger correlation coefficient (p < 0.0005) and slope (p < 0.0005) that characterized the regression of Audio with respect to Baseline frames.

### 3.2 Pupil averaging and adaptation

For each individual subject, we conducted separate averaging procedures for the first and the second block of 15 trials, as well as for all the remaining traces (third block). We evaluated the significance of the difference between the Audio and Baseline traces for each of the three blocks of trials. This analysis was performed to assess whether the restriction of the averaging to the initial trial, would have enhanced the chance to obtain significant PDR by discarding traces obtained while the subject was adapted to the stimulus. However, only one subject (in LL condition), more than those displayed in Table 2, was found by restricting the averaging to the initial block of trial. Consistently with an adaptation to sound stimulation, no subject showed significant PDR in the second block of trials. Surprisingly, in the third block of trials, four subjects showed again a pupil responsiveness to sound.

**Fig. 6:**
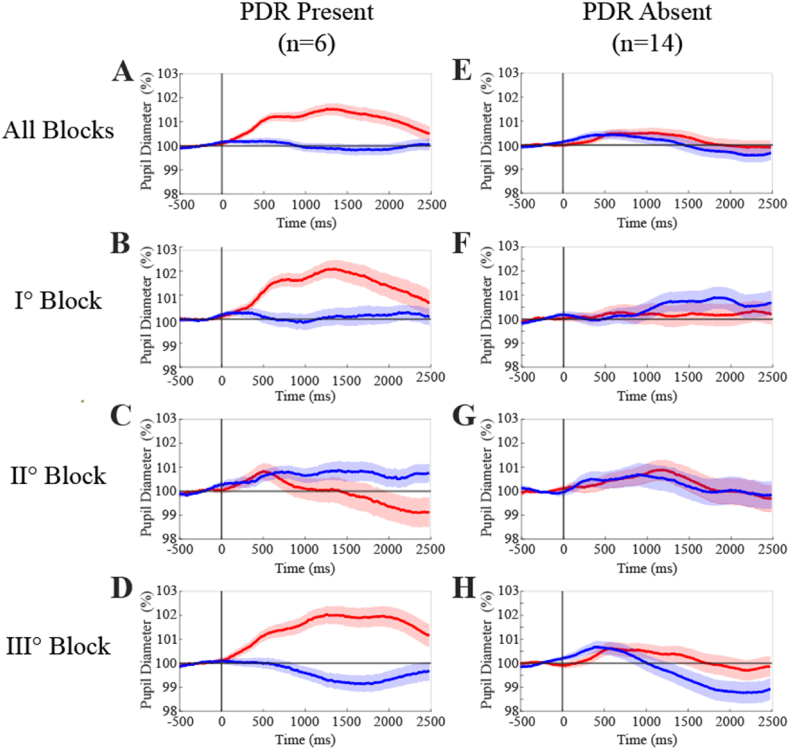
**Grand average of Audio and Baseline pupil traces for subjects with and without significant PDRs to sound.** The grand average of Audio and Baseline traces was separately performed for the cases showing presence (n = 6, A-D) or absence (n = 14, E-H) of significant PDRs to sound. Data relative to all traces are shown in A and E. Those relative to the first, second and third blocks of data can be appreciated in B/F, C/G and D/H, respectively. Audio and Baseline traces correspond to red and blue lines, respectively. The colored regions around average traces correspond to SE values.(For interpretation of the references to color in this figure legend, the reader is referred to the Web version of this article.)

Therefore, when we restricted the averaging procedure to single block trials, the number of cases with significant PDRs rose from 5 to only 6. Two subjects belonged to the HL and four to the LL group, corresponding to 20 % and 40 % of the sample size (n = 10 in both instances). The grand averages of the PDRs of these six cases evaluated for each of the three blocks of traces are displayed in Fig. 6A (all blocks), 6B (block I), 6C (block II), and 6D (block III). Significant Audio-Baseline differences were observed in the 0.5–2.0 s interval for the first and third blocks of trials (p < 0.001 in both instances), but not for the second block. Additionally, it is noteworthy that no significant Audio-Baseline differences were detected when we performed the grand average on the remaining 14 cases, whatever block was considered (Fig. 6E-H).

## 4 Discussion

This study aimed to assess the potential of detecting PDR in individual normal subjects exposed to a supra threshold standard audiometric stimulus consisting of a pure tone at a single frequency. The establishment of a protocol that guarantees a very high chance (near to 100 %) of retrieving PDR when perception occurs in normal subjects is a prerequisite for exploring the feasibility of utilizing PDR recordings as a substitute for conventional audiometry in subjects who cannot communicate, or as an alternative to the relatively cumbersome electrophysiological auditory tests.

It can be asked to what extent the PDR, which is a measure of the activation of the arousal system [29], can be utilized as an indicator of perception [30]. It has been shown that a PDR occurs when visual stimuli of threshold intensity are perceived, while this is not the case when the stimulus is undetected [30]. For this reason pupillometry has been utilized as a covert measure of visual stimuli perception [31]. A positive relation between pupil diameter and perception has been also documented for tactile [32] and acoustic stimuli [17]. It appears, therefore, that such a test can potentially assess hearing capability in people who are unable to communicate but show a normal level of arousal, provided that the correspondence between PDR and perception is documented at increasing stimulus amplitude starting from threshold. Yet, when arousal is present, but awareness is questionable, such as it may occur in dementia, PDRs can still be detected [[23], [24], [25]], thus indicating the subject's nervous system can be entrained by the acoustic stimulus.

In the present study the presence of PDRs was verified by a rigorous statistical procedure, to ensure reliability and avoid false positives caused by occasional fluctuations in pupil size following auditory stimulation. The results obtained clearly demonstrate that there is no direct one-to-one correlation between the presentation of a clearly audible, supra-threshold stimulus of a typical standard audiometric procedure sound and the detection of a PDR at the individual subject level. So, perception may easily occur in the absence of a PDR. Moreover, to achieve our low percentage of significant PDRs, we had to test the subjects for 12–18 min, that is a rather long period of time for a hearing test. Therefore, modifications to the audiometric evaluation procedure are necessary to effectively employ PDR as an indicator of hearing capability.

It can be wondered whether the different autonomic responsiveness of the individual subjects to sound could be related to differences in the pre-stimulus values of pupil size. Unfortunately, the technique used in the present experiment, although well suited to capture within-subject pupil size variations, did not allow a fine between subject comparison of the pupil diameter (see Methods). However, a within-subject analysis separately performed in each of the subjects displayed in Table 2 always showed a significant, negative correlation between the PDR and the pre-stimulus pupil size value. This correlation could be sometimes observed also when the stimulus was absent and equally spaced frames were randomly selected, reflecting a higher chance of having a pupil diameter decrease following a period of pupil dilatation and vice versa. However, the correlation was significantly stronger and steeper for Audio than Baseline frames (Fig. 5). These data suggest that an increase in pupil size baseline value, related to spontaneous changes in alertness, tends to decrease the PDR: this would be expected when the level of the tonic Locus Coeruleus activity is high [32]. So, a possibility exists that the individual variability in PDR is related to between-subjects differences in pre-stimulus pupil size values that cannot be assessed with the technique utilized in the present study.

The weak PDR may result from a low level of arousal induced by the constancy of stimulus amplitude and frequency, which causes adaptation of the response [17]. In the present experiments, the same sound was presented 120 times, every 4 s for 8 min, while previous investigations showed adaptation in the first 30 trials even with an interstimulus interval of 8 s [17]. Repeating the same sound every 4 s or so is necessary to keep test duration short for a clinical application, but undoubtedly it speeds up the adaptation. Indeed, limiting the averaging to the initial block of trials revealed only a significant PDR more than the five observed by averaging all the trials. The adaptation of PDR was confirmed in this report since no significant PDR was observed in the second block of trials. Finally, a recovery of the response was observed in the third block of trials, possibly due to a renewed engagement in the task and an increase in attention levels, which counteracted the habituation effect to the repeated identical stimulus.

Finally, it is worth of noting that a low luminance level is the most favorable condition to detect the PDR. Indeed, the grand average of audio traces exhibited a significant post-stimulus difference compared to baseline traces in low luminance conditions, which was not the case in experiments conducted under high environmental lighting. This finding aligns with previous research indicating that PDRs in response to acoustic stimuli are enhanced in low-light conditions. The lower level of luminance corresponds to reduced parasympathetic influence on pupil size [33,34], thus suggesting that the sympathetic system plays a dominant role in triggering PDR associated with sound presentation [34]. The increased PDR in a low environmental lighting, i.e., with a larger pupil size, could be attributed to a reduction of visual distractors which allows a focus of attention on the acoustic stimulus. In contrast, during the random pupil size fluctuations occurring during the audiometric test, the highest pre-stimulus values are associated with the lowest PDR amplitudes. This finding is expected to occur when the basal level of tonic Locus Coeruleus activity is high [32]. So, the effects of pre-stimulus pupil diameter on PDR are different according to the source of pupil size modifications.

The present data indicate that, to use the PDR as a hearing ability test, new testing procedures must be implemented. Previous investigators have used stimuli of randomly changing frequency and amplitude, reporting individual PDR in normal subjects [17]. Although the percentage of retrieved PDR to supra-threshold stimuli has not been given by the authors, it is conceivable that the use of this technique may give rise to more consistent and frequent responses. Additionally, subjects can be provided with more precise instructions to restrict gaze movement, rather than merely asking them to "look forward." This instruction may allow for variations in gaze direction between areas with different luminance levels, which can affect pupil size [35] and changes in the fixation depth [36], making the detection of PDRs in response to sounds more challenging. Lastly, the detection of PDRs may be enhanced by averaging more trials, thereby extending the duration of the entire test. A completely new path can be taken, that of using sounds associated with specific events, which have a positive value and evoke an emotional response.

In conclusion, recording PDR in response to sound as reliable indicator of hearing capability is not achieved by a standard audiometric procedure and further attempts to utilize the PDR as a test of hearing perception in populations of subjects who are unable to communicate with clinical operators, require drastic modifications of the testing procedure.

## CRediT authorship contribution statement

**Maria Paola Tramonti Fantozzi:** Writing – review & editing, Writing – original draft, Visualization, Investigation, Formal analysis, Data curation. **Antonino Crivello:** Writing – review & editing, Writing – original draft, Visualization, Validation, Supervision, Software, Resources, Project administration, Methodology, Investigation, Funding acquisition, Formal analysis, Data curation, Conceptualization. **Davide La Rosa:** Writing – review & editing, Writing – original draft, Visualization, Validation, Software, Methodology, Investigation, Formal analysis, Data curation, Conceptualization. **Mario Milazzo:** Writing – review & editing, Writing – original draft, Visualization, Validation, Supervision, Software, Resources, Methodology, Investigation, Formal analysis, Data curation, Conceptualization. **Serena Danti:** Writing – review & editing, Writing – original draft, Visualization, Validation, Investigation, Formal analysis, Conceptualization. **Vincenzo De Cicco:** Writing – review & editing, Writing – original draft, Validation, Methodology, Investigation, Data curation. **Paolo Orsini:** Writing – review & editing, Writing – original draft, Visualization, Software, Investigation, Formal analysis, Data curation. **Diego Manzoni:** Writing – review & editing, Writing – original draft, Visualization, Validation, Supervision, Software, Resources, Methodology, Investigation, Formal analysis, Data curation, Conceptualization. **Francesco Lazzerini:** Writing – review & editing, Writing – original draft, Visualization, Validation, Investigation, Formal analysis, Data curation. **Rachele Canelli:** Writing – review & editing, Writing – original draft, Visualization, Validation, Investigation, Formal analysis, Data curation. **Giacomo Fiacchini:** Writing – review & editing, Writing – original draft, Visualization, Validation, Data curation. **Luca Bruschini:** Writing – review & editing, Writing – original draft, Validation, Supervision, Resources, Methodology, Investigation, Funding acquisition, Formal analysis, Data curation, Conceptualization.

## Data availability statement

Raw data collected in this study is available at https://zenodo.org/doi/10.5281/zenodo.10497437.

## Declaration of competing interest

The authors declare that they have no known competing financial interests or personal relationships that could have appeared to influence the work reported in this paper.
